# Binary Mixture of Neonicotinoid–Pyrethroid Insecticide: Impact on Survival, Cognitive Learning, and Memory in *Apis mellifera jemenitica*

**DOI:** 10.3390/biology14020147

**Published:** 2025-01-30

**Authors:** Mohamedazim I. B. Abuagla, Javaid Iqbal, Hael S. A. Raweh, Abdelsalam S. A. Abdelaziz, Abdulaziz S. Alqarni

**Affiliations:** 1 Department of Plant Protection, College of Food and Agricultural Sciences, King Saud University, P.O. Box 2460, Riyadh 11451, Saudi Arabia; mabuagla@ksu.edu.sa (M.I.B.A.); jiqbal@ksu.edu.sa (J.I.); hraweh@ksu.edu.sa (H.S.A.R.); 2 Key Laboratory of Forestry and Grassland Administration on Forest Ecosystem Protection and Restoration of Poyang Lake Watershed, College of Forestry, Jiangxi Agricultural University, Nanchang 330045, China; asa26@stu.jxau.edu.cn

**Keywords:** combination index, honey bee, insecticide mixture, learning and memory formation, synergistic effects, topical and oral application, toxicity

## Abstract

The impact of agrochemicals on pollinators, especially honey bees, has drawn significant attention due to its critical implications for worldwide food stability and ecosystems. Given the potential threat of insecticides to honey bees, bees may encounter multiple insecticides simultaneously during foraging. This study investigated the toxic effect of an insecticide mixture (IM) containing acetamiprid (neonicotinoid) and deltamethrin (pyrethroid) on the survival and cognitive appetitive performance of *Apis mellifera jemenitica,* a vital native pollinator in arid regions of Saudi Arabia. The lethal concentration (LC_50_) was determined by assessing bees’ mortality rates following exposure to IM through topical and oral routes. Significant bee mortality occurred at 4–48 h post treatment with IM through both exposure routes, showing a trend of increased mortality with higher IM concentrations compared to the control bees. Throughout all tested times, topical exposure proved relatively more effective, resulting in significantly greater bee mortality compared to oral exposure to IM. Food intake declined progressively with rising IM concentrations during oral exposure. The LC_50_ values of IM at 24 h after treatment were 12.24 ppm for topical and 10.45 ppm for oral exposure. The corresponding LC_10_, LC_20_, and LC_30_ values were 3.75 ppm, 5.63 ppm, and 7.54 ppm for topical exposure and 2.45 ppm, 4.04 ppm, and 5.78 ppm for oral exposure, respectively. The combination index (CI) revealed a synergistic effect (0.43) for topical exposure and antagonistic effects (1.43) for oral exposure, highlighting differential toxicity dynamics. IM exposure significantly impaired cognitive acquisition and memory reinforcement in honey bees, as demonstrated through behavioral assays, indicating potential neurotoxic effects. Learning and memory formation significantly declined at 2, 12, and 24 h after exposure to sublethal concentrations of IM through both topical and oral routes. Thus, evaluating the interactive impact of multiple pesticides on bees’ health and cognitive function is essential, particularly in regions where diverse agrochemicals are routinely utilized.

## Simple Summary

Honey bees are vulnerable to multiple stressors, including pesticide toxicity during foraging. They can be exposed to pesticides through various routes, such as oral ingestion of contaminated pollen, nectar, or water, and contact with pesticide residues on plants and environmental surfaces. Beyond individual pesticide poisoning, the combined effects of pesticide mixtures—whether synthetic or naturally occurring—can be lethal to bees, even at sublethal concentrations. This study investigates the effects of a binary mixture of two commonly used pesticides, acetamiprid (neonicotinoid) and deltamethrin (pyrethroid), on the survival and cognitive functions of the native Saudi Arabian honey bee species, *Apis mellifera jemenitica*. The insecticide mixture caused significantly higher mortality and impaired learning and memory formation, as assessed through lethal concentration analysis and olfactory learning paradigms via oral and topical exposure routes. These findings enhance our understanding of synergistic and antagonistic interactions between pesticides and highlight the need for cautious pesticide use to protect pollinator health and ecosystem stability.

## 1. Introduction

Pollination is essential for sustaining plant, animal, and human life and food security [1,2,3]. Approximately 75–80% of cultivated crops and flowering plants rely on pollinators for reproduction and contribute to enhance ecosystem diversity by facilitating the pollination of a variety of plants [4,5,6,7]. Honey bees, as the most prevalent and valuable crop pollinator, play a crucial part in this process [8,9,10,11,12]. Recent studies and FAO publications have reported a noticeable decline in pollinator numbers [13,14].

Honey bees, in particular, are declining at an alarming rate, impacting agriculture, biodiversity, and the ecosystem [15,16]. Several factors are recognized as driving the decline of pollinators, such as habitat damage, climate change, loss of biodiversity, modern agricultural practices, competition from non-native species, parasites and diseases, and exposure to agrochemicals, including insecticides [17,18,19,20,21,22]. These factors negatively impact pollinators, including honey bees, potentially leading to rapid population decline or colony collapse disorder (CCD) phenomena [23]. CCD was first observed in 2006, following the sudden decline of approximately one-third of bee colonies in North America [16,24].

In modern agriculture, protecting crops from insect pests heavily relies on pesticide use, which can inadvertently expose bees to toxic chemicals during foraging on flowering plants. Their widespread use has significant implications for pollinators, particularly honey bees [25]. These chemicals primarily accumulate in the soil, air, and organisms within ecosystems [26,27]. Insecticides have a great deal of toxicity, depending on many factors, including their type, mode of action, toxicity profile and concentration, time and mode of application, and persistence in the environment [28,29]. The potential risks to honey bee populations fluctuate depending on the levels and patterns of their exposure to chemicals [30,31].

Honey bees are crucial for agriculture productivity and biodiversity [25]. Globally, beekeepers report significant declines in the bee population due to synthetic pesticides, which could reduce bee survival rates by nearly fivefold [32,33]. Honey bees frequently encounter pesticides, potentially through multiple pathways, including direct contact with treated plants, contaminated nectar, pollen, and water sources, and aerial spray drift during foraging [34,35,36]. Exposure to pesticides, including insecticides, herbicides, and fungicides, can lead to both acute and sublethal effects in honey bees [37], leading to immediate mortality and negatively impacting bee population and behavior [38,39]. Insecticide concentration is a determinant factor, as sublethal concentrations can disrupt physiological and behavioral functions, while higher concentrations may lead to acute toxicity and mortality [40,41].

Among the various pesticides, neonicotinoids and pyrethroids target the insect nervous system and sodium channels in nerve cells, respectively, posing potential harm to pollinators [42,43]. These broad-spectrum insecticides, extensively used in agriculture, public health, and household insect control [44,45,46], have been shown to negatively affect honey bees’ health in multiple ways [47,48,49,50]. Neonicotinoids, present in pollen and nectar, vital food sources for bees, can reduce honey bee performance [51,52] and impair foraging, navigation, and communication [53]. Pyrethroids, which are neurotoxic with a rapid knockdown effect, can adversely affect fecundity, orientation, communication, foraging activity, and other aspects of honey bee behavior [47,54].

In addition to individual pesticides, honey bees are often exposed to a complex mixture of different pesticides in the field through contact and oral ingestion routes. Such combinations, whether from blended formulations or environmental mixing, may interact to produce additive, synergistic, or antagonistic effects on bee health, behavior, physiology, and immune responses, further disrupting colony dynamics, impair pollination services, and ultimately threaten the honey bee population [5,30,55,56]. The synergistic effects of multiple chemicals are a critical concern for honey bees’ health, as their combined effects may intensify their negative impacts. Thus, understanding the interactions between various insecticides is vital for assessing their ecological consequences. By identifying the type of interaction, researchers can develop strategies to mitigate risks and ultimately protect these essential pollinators. This knowledge is crucial for conducting the effective risk assessment of potential pesticide interactions and ensuring the sustainability of both pollinator populations and agricultural ecosystems.

Despite considerable research on individual pesticides, investigations into the interactive toxicity of the combined mixture of neonicotinoid and pyrethroid on honey bees remain limited. This study addresses this research gap by evaluating the potential detrimental effects of a binary mixture of deltamethrin (a pyrethroid) and acetamiprid (a neonicotinoid) on the survival and cognitive performance of honey bees under controlled laboratory conditions. These findings can help researchers prioritize conservation efforts to safeguard pollinators in regions like Saudi Arabia.

In Saudi Arabia, the expansion of agriculture and a rising food demand have led to increased pesticide use [57]. Synthetic pesticides, such as deltamethrin and acetamiprid, registered by the Saudi Food and Drug Authority, are commonly used to control agricultural pests and public health insects [58]. The extensive use of pesticides poses significant risks to pollinators, especially honey bees [41], with pesticide residues in agricultural products raising concerns about contamination and ecological impact [59,60]. Beekeeping is a vital component of Saudi Arabia’s agricultural sector, significantly contributing to biodiversity, enhancing rural livelihoods, and boosting crop production through pollination, as well as providing valuable bee products, including honey [61,62,63]. The native honey bee, *Apis mellifera jemenitica*, is adapted to extreme hot and arid conditions, making it crucial for pollination and biodiversity [64,65]. However, there are potential threats to them from extensive pesticide use, which could jeopardize both bee survival and the sustainability of beekeeping. This study, therefore, extends prior research on the individual impacts of these pesticides by investigating their combined effects on *A. m. jemenitica*.

## 2. Materials and Methods

### 2.1. Bee Species and Bee Collection

*Apis mellifera jemenitica* specimens, a native subspecies in Saudi Arabia, were reared at King Saud University’s educational farm (24°44′14.2″ N 46°37′09.9″ E). This subspecies is prevalent and dominant across the Arabian Peninsula, including Saudi Arabia [61,66]. Five bee colonies were maintained following standard beekeeping practices without the use of pesticide, ensuring that they remained free of pathogen infestations and insecticide exposure. Forager bees (80–100) returning from foraging were evenly collected from the entrance of all colonies using a fine brush [41,65], maintained in wooden cages (15 × 15 × 5 cm: L × H × W) and acclimatized for 2 h in the laboratory at 25 °C prior to subsequent analyses [47]. The acclimatized bees were then uniformly allocated to each treatment group for all analyses.

### 2.2. Preparation of Insecticide Mixture (IM)

Pyrethroid (deltamethrin; Klash^®^ 25 EC, 25 g/L, Astrachem, Astra Industrial Complex Co., Ltd., Dammam, Saudi Arabia) and neonicotinoid (acetamiprid; Cetam^®^ 20SL, Al-Burj Agrivet Pesticide Manufacturing Co., Ltd., Amman, Jordan and distributed by Saudi United Fertilizer Co., Jeddah, Saudi Arabia) were mixed in an LC_50_: LC_50_ ratio [67] (36.16:160.33 ppm for topical and 32.53:12.76 for oral ingestion) to prepare an insecticide mixture (IM). These LC_50_ values corresponding to topical and oral exposure were adopted as reported in our previous studies for deltamethrin [47] and acetamiprid [50].

### 2.3. Toxicity Analyses of IM

To estimate the toxicity of the IM on bee mortality, the insecticide treatment was administrated through two exposure routes: topical application on the thorax and oral ingestion. Bee mortality data were recorded at 4, 12, 24, and 48 h post treatment, and the lethal concentrations (LC_50_ and LC_90_) were determined. The Abbott formula was used to calculate the lethal concentration (LC_50_) and the sublethal concentrations (LC_10_, LC_20_, and LC_30_), using the corrected bee mortality after 24 h in response to the serial dilutions of the IM and the control.

#### 2.3.1. Topical Exposure of IM

The serial dilutions of IM (98.25, 49.50, and 24.60 ppm) were prepared in acetone from the IM stock solution (196.50 ppm) for topical application. A control (acetone) was also included in the analysis for topical application. Ten bees (randomly taken from wooden cages) for each IM dilution per replication were immobilized on ice for 3–5 min, and 1 µL of each dilution of IM was individually applied to the dorsal side of the thorax of each bee using a micropipette [68]. The treated bees were then transferred to the plastic containers (diameter: 12 cm; height: 10 cm) [47]. Treated group of bees from each dilution kept in individual plastic containers were provided with two separate syringes (5 mL) of 50% sucrose solution and water to ensure an adequate food supply. The bees were incubated in an incubator (LIB-060M, Lab Tech, Daihan Lab Tech Co., Ltd., Namyangju-City, Gyeonggi–Do, Republic of Korea) at 25 ± 2 °C and 60 ± 10% RH, and mortality data were recorded at specific post-treatment time periods (4, 12, 24, and 48 h) to calculate the mortality rates for the tested IM dilutions. The experiment was conducted in four replicates, with each IM dilution consisting of ten bees per replication, and each dilution housed in a separate plastic cage, resulting in a total of forty bees across four replications for each treatment.

#### 2.3.2. Oral Exposure of IM

The IM stock solution (45.30 ppm) was used to prepare serial dilutions (22.65, 11.30, and 5.65 ppm) in a 50% (*w/v*) sucrose solution for oral feeding, with a sucrose solution serving as the control. The forager bees in the wooden cages were starved for 2 h in the laboratory at 25 °C to induce substantial starvation. Ten starved bees (randomly taken from the wooden cages) for each IM dilution per replication were transferred to the individual plastic containers, and each plastic container was provided with 200 μL of IM-tainted sucrose solution corresponding to each dilution [69]. A separate group of bees was given only the sucrose solution as the control. The bees were maintained in the dark in an incubator (LIB-060M, Lab Tech, Daihan Lab Tech Co., Ltd., Namyangju- City, Gyeonggi–Do, Republic of Korea) set at 25 ± 2 °C and 60% ± 10% RH, and were allowed to feed for 4 h. The remaining IM-contaminated food was removed from the plastic containers. Food consumption was assessed by measuring the quantity of food before and after a 4 h feeding period for each group. The evaporation of the IM-tainted solution was accounted for by measuring the evaporated quantity and subtracting it from the total consumption for each group. The data were then used to calculate the percentage of food consumption. After the 4 h feeding period, the bees were left without food for 1 h to aid the digestion of the ingested food. Afterwards, the bees were provided a 50% sucrose solution and water ad libitum to satisfy their nutritional requirements and ensure survival throughout the experiment [70]. Mortality data were recorded at 4, 12, 24, and 48 h post treatment to assess the impact of different IM dilutions on bee mortality. The experiment was conducted in four replicates, with each IM dilution consisting of ten bees per replication, and each dilution housed in a separate plastic cage, resulting in a total of forty bees across four replicas for each treatment.

#### 2.3.3. Combination Index (CI) for Mixture Toxicity Analysis

Toxicity interactions were determined based on the LC_50_ values for each insecticide and their insecticide mixture (IM) [67,71]. The binary interaction between two insecticides was assessed through combination index (CI) analysis to evaluate synergistic, antagonistic, and additive effects using the following formula [67,72,73].

$${CI}_{50}=\frac{{LC}_{50}^{M}}{{LC}_{50}^{A}}+\frac{{LC}_{50}^{M}}{{LC}_{50}^{B}}+\left(\frac{{LC}_{50}^{M}}{{LC}_{50}^{A}}\times \frac{{LC}_{50}^{M}}{{LC}_{50}^{B}}\right)$$

Here, LC_50_^A^ and LC_50_^B^ represent the median lethal concentrations of insecticides A and B, respectively, when each is tested alone. LC_50_^M^ is the median lethal concentrations of the insecticide (A + B) mixture. The value of CI represents the respective interaction patterns of IM: an additive effect is indicated by a value of 1, an antagonistic effect by values greater than 1, and a synergistic effect by values less than 1.

### 2.4. Olfactory Cognitive Test for Learning and Memory Formation

Adult foragers (100–120) were captured randomly at the hive entrances using wooden cages and a fine brush. Following 3–5 min of chilling on ice, the bees were harnessed using the procedures established in prior research [41,74]. The bees were fed with a 0.5 M sucrose solution and kept overnight in a dark and humid environment (25 ± 2.0 °C and 50 ± 10% RH). Afterwards, the bees were motivated using a 0.5 M sucrose solution by touching their antennae without providing feeding, and non-responsive bees were discarded. Only those bees that successfully demonstrated motivation by extending their proboscises were used for subsequent IM treatment through topical or oral application in separate experiments. Four replicas were conducted for each IM concentration, using twenty bees for each concentration within a single iteration [47,50].

For topical application, 1 µL of each sublethal concentration (LC_10_ = 3.75 ppm, LC_20_ = 5.63 ppm, and LC_30_ = 7.54 ppm) of IM was applied on the dorsal side of the thorax of each bee using a micropipette, one hour prior to the learning trials. The control bees were treated with 1 µL of acetone alone. For oral application, bees in different groups were fed 1 µL of each sublethal concentration (LC_10_ = 2.45 ppm, LC_20_ = 4.04 ppm, and LC_30_ = 5.78 ppm) of IM one hour prior to the learning trials. The control bees received only 1 µL of a 50% sucrose solution without acetone. Learning trials were conducted using a classical olfactory associative learning protocol, in which each conditioning trial involved pairing an odor stimulus (clove oil) as the conditioned stimulus (CS) with a reward stimulus (1M sucrose) as the unconditioned stimulus (US) [41,65,74,75]. Each bee was trained with three learning trials at 10 min intervals. Memory retention was tested at different times (2, 12, and 24 h) after the learning trials using only the odor stimulus (CS). The proboscis extension response (PER) was noted to assess the learning and memory capabilities of honey bees [75]. Bees extending their proboscis were marked as positive, while non-responders were marked negative. The percentage PER was calculated for both learning and memory tests. To ensure the survival and nutritional needs, harnessed bees were provided with a 0.5 M sucrose solution every 4 h during the experimental period [41,65].

### 2.5. Data Analysis

The Abbott formula, as outlined by Finney [76], was used to correct the bee mortality [69,70], and lethal and sublethal concentrations of IM were determined with the LdP Line software (https://www.ehabsoft.com/ldpline/) [77]. Data on bee mortality and food consumption during oral feeding were analyzed using ANOVA, with the means separated by a least significant difference (LSD) post hoc test using the SAS 9.2 software. Using nonparametric tests (Fisher’s exact/Chi-square (χ^2^) at (*p* ≤ 0.05)), the PER values during the cognitive tests were analyzed.

## 3. Results

The insecticide mixture (IM) comprising acetamiprid and deltamethrin demonstrated significant bee mortality and induced notable alterations in the behavioral performance of forager bees, specifically in learning and memory formation.

### 3.1. Impact of Topical IM Application on Bee Mortality

**Figure 1:**
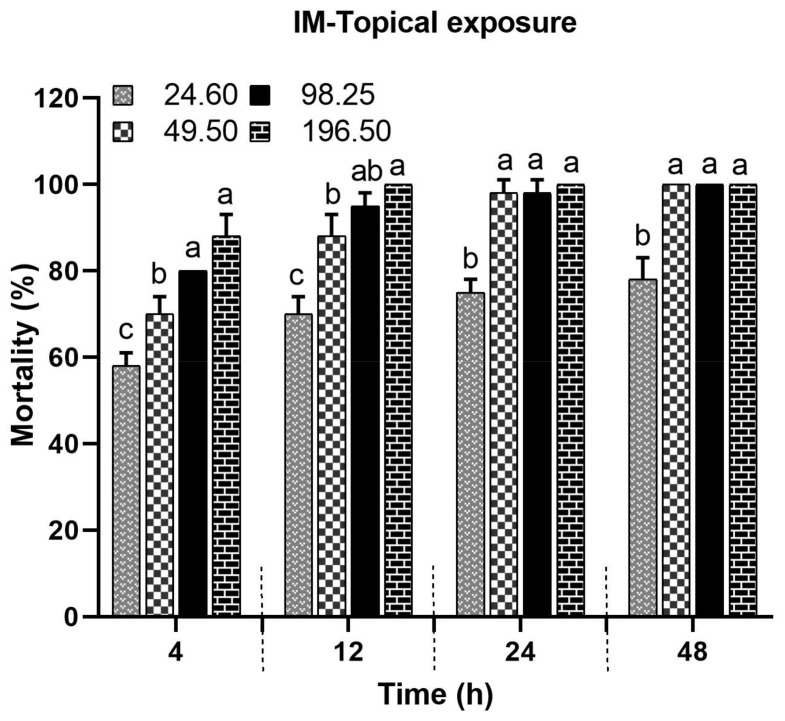
Bee mortality after topical application of IM (mean ± SE). Different letters across tested times indicate significant differences (*p* ≤ 0.05).

A significant difference (F(4) = 946.66; *p* < 0.0001) in bee mortality was observed after topical exposure to the tested concentrations of IM (196.50, 98.25, 49.50, and 24.60 ppm) at different post-treatment times (4, 12, 24, and 48 h). Additionally, bee mortality significantly varied across the post-treatment times (F(3) = 40.55; *p* < 0.0001). All tested concentrations resulted in a bee mortality above 50% within 4 h of treatment, and bee mortality increased proportionally with rising IM concentrations. One hundred percent mortality was recorded at three concentrations (196.50, 98.25, and 49.50 ppm) at both 24 h and 48 h post treatment. The lower concentration (24.60 ppm) also resulted in substantial mortality, exceeding 50% across all tested times (Figure 1). The control bees exhibited no mortality, with significant differences compared to IM-treated bees (Table S1).

#### 3.1.1. Impact of Oral IM Application on Bee Mortality

**Figure 2:**
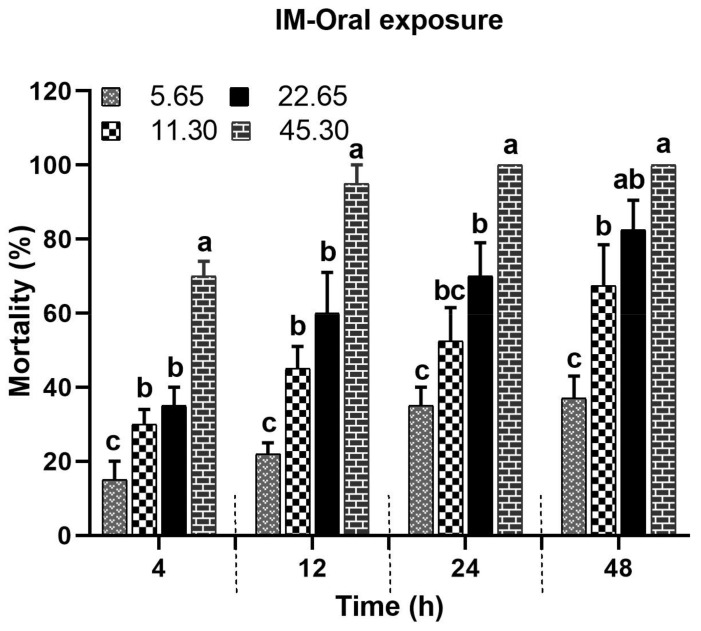
Bee mortality after oral ingestion of IM (means ± SE). Different letters across tested times indicate significant differences (*p* ≤ 0.05).

The oral administration of the IM concentrations (45.30, 22.65, 11.30, and 5.65 ppm) showed significant differences in the mortality percentage of the tested bees compared to the control group (F(4) = 141.63; *p* < 0.0001). Additionally, bee mortality significantly varied across post-treatment times (F(3) = 20.84; *p* < 0.0001). The mortality increased with an increase in the IM concentration. The highest mean mortality (70, 95, 100, and 100%) was observed with 45.30 ppm, whereas the lowest mean mortality (15, 22, 35, and 37%) was observed with 5.65 ppm at 4, 12, 24, and 48 h, respectively (Figure 2). The control bees exhibited no mortality, revealing the significant effects of IM on the treated bees (Table S2).

#### 3.1.2. Food Consumption After Oral Ingestion of IM

**Figure 3:**
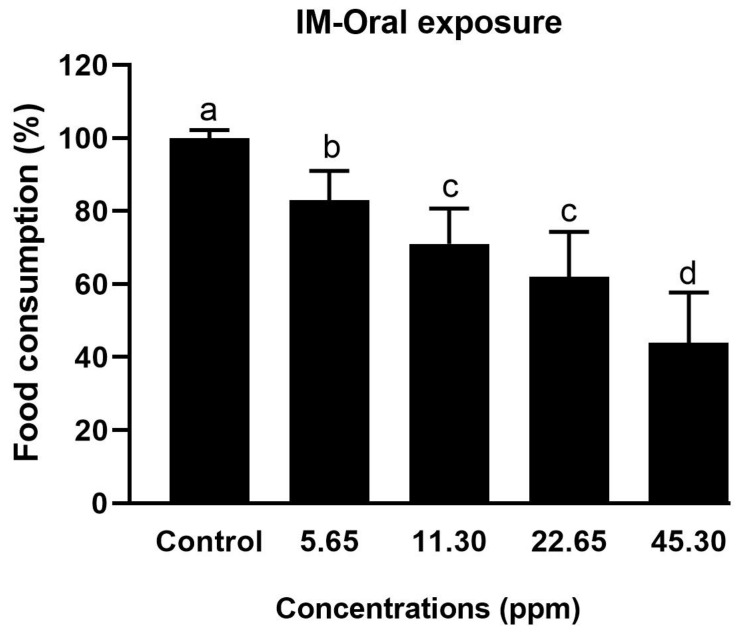
Food consumption (%) of *A. m. jemenitica* under the oral administration of the insecticide mixture. Similar letters denote no significant difference among the tested concentrations at *p* ≤ 0.05.

The oral administration of IM concentrations (45.30, 22.65, 11.30, and 5.65 ppm) resulted in a significant reduction (F(4) = 5.40; *p* = 0.0067) in food consumption compared to the control. The food consumption was concentration-dependent and decreased with an increase in the concentration of IM. The lowest food consumption (44%) was observed with 45.30 ppm, and the highest food consumption was in the control (100%). Food consumption was gradually reduced (100, 83, 71, 62, and 44%) with the tested concentrations (0, 5.65, 11.30, 22.65, and 45.30 ppm, respectively) of IM (Figure 3). Therefore, a higher concentration of IM in the food resulted in a lower food consumption rate by the bees (Table S3). Thus, the oral intake of a high concentration (45.27 ppm) of IM led to the decreased intake of food, but it was enough to cause the highest mortality (Figure 2).

#### 3.1.3. Combination Index of Insecticide Mixture

The toxicity binary interactions of insecticides (deltamethrin–acetamiprid) in the insecticide mixture (LC_50_: LC_50_) were assessed by calculating the combination index (CI) to evaluate antagonistic, synergistic, and additive effects following both topical and oral exposure routes. For topical exposure, the CI was 0.43, indicating a synergistic effect, while for oral exposure, the CI was 1.40, suggesting an antagonistic effect (Table 1).

#### 3.1.4. Comparison of Bee Mortality: IM vs. Individual Insecticide

**Figure 4:**
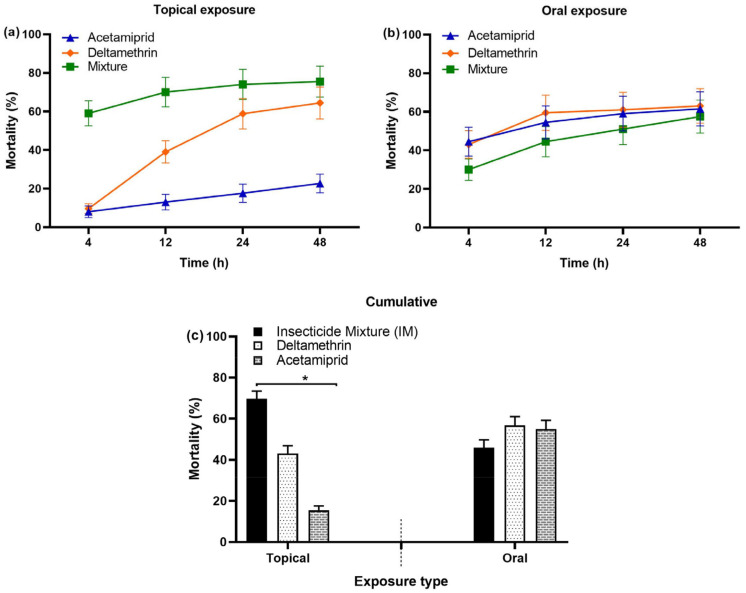
Comparison of bee mortality in response to the mixture of deltamethrin–acetamiprid insecticide: (**a**) topical exposure, (**b**) oral exposure, and (**c**) cumulative comparison. Asterisk (*) indicate significant differences (*p* ≤ 0.05) among treatments following two distinct routes of topical and oral exposure.

The IM (deltamethrin–acetamiprid) resulted in a higher bee mortality following topical exposure compared to the individual application of deltamethrin and acetamiprid alone (Figure 4a,c). Oral exposure to IM led to a relatively lower, but non-significant, mortality compared to each individual insecticide (Figure 4b,c). For the comparative analysis between the IM and each insecticide, bee mortality data for acetamiprid and deltamethrin were sourced from our earlier publications [47,50], conducted under identical environmental conditions to those described in the current study. The IM experiments were performed during the same experimental period, immediately following those with the individual insecticides [47,50].

#### 3.1.5. Lethal and Sublethal Concentrations

The lethal concentrations (LC_50_/LC_90_) of IM for topical and oral exposure were established 24 h after the treatment. The LC_50_ values were found to be 12.24 ppm and 10.45 ppm for topical and oral exposure, respectively (Table 2). Sublethal concentrations (LC_10_, LC_20_, and LC_30_) of IM were recorded as 3.75, 5.63, and 7.54 ppm after topical exposure and 2.45, 4.04, and 5.78 ppm for oral exposure 24 h post treatment (Table 2).

#### 3.1.6. Comparative Analysis: Topical vs. Oral Exposure for IM-Induced Bee Mortality

**Figure 5:**
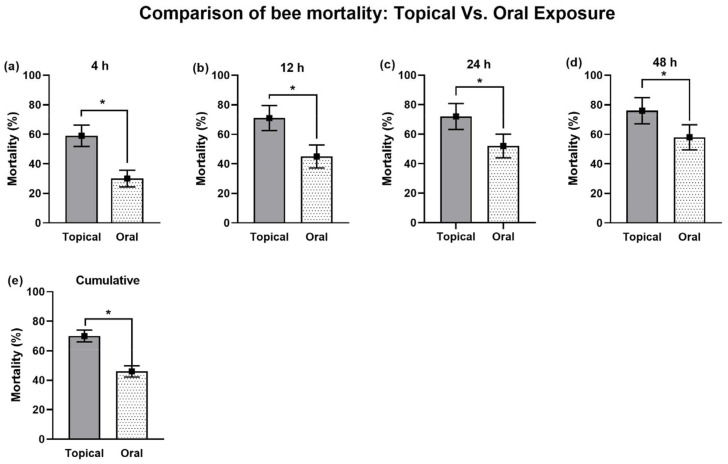
Analysis of mortality in *A. m. jemenitica* based on different exposure routes to IM at (**a**) 4 h, (**b**) 12 h, (**c**) 24 h, and (**d**) 48 h post treatment and (**e**) cumulative mortality. Asterisk (*) indicate significant differences observed at each tested time point (*p* ≤ 0.05, T-test).

Topical exposure to IM exhibited a significantly higher mortality compared to oral exposure at all tested post-treatment times (4, 12, 24, and 48 h) (Figure 5a–d). The overall bee mortality, regardless of the concentration and the time, also demonstrated a similar trend, where mortality after topical exposure (70%) was significantly greater than that after oral exposure (46%) (Figure 5e).

### 3.2. Honey Bee Olfactory Responses

The olfactory cognitive ability, learning, and memory retention of honey bees were assessed at different times (2, 12, and 24 h) post treatment with sublethal concentrations of IM after topical application and oral ingestion.

#### 3.2.1. Topical Exposure of IM and Associative Learning

**Figure 6:**
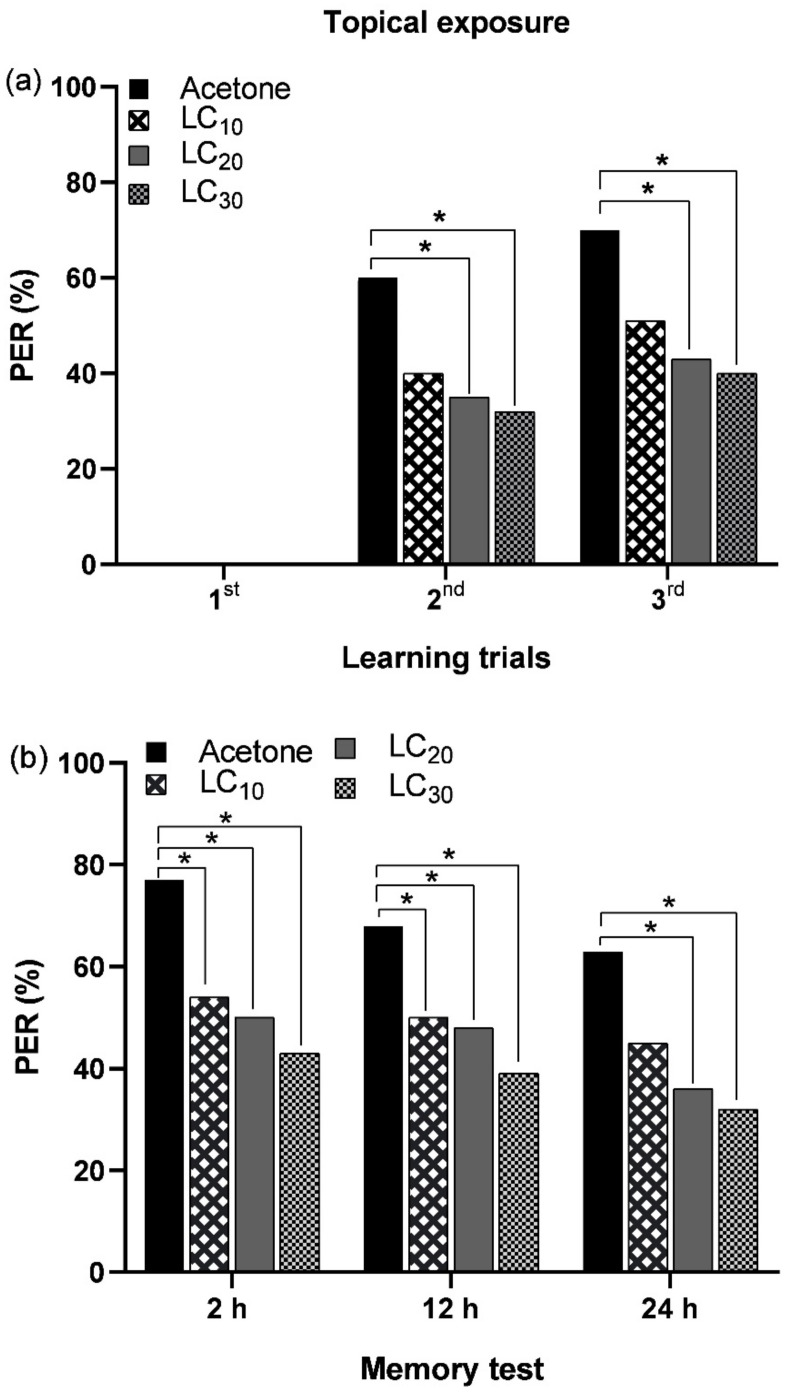
Proboscis extension response (PER) of bees during (**a**) learning trials and (**b**) memory tests following topical exposure to various sublethal concentrations of IM. Asterisk (*) indicate a significant difference between the control and IM-treated bees (Fisher’s exact test/χ^2^ test; * *p* < 0.05).

The topical application of sublethal concentrations of IM (LC_10_ = 3.75 ppm, LC_20_ = 5.63 ppm, and LC_30_ = 7.54 ppm) negatively affected the olfactory learning and memory formation of *A. m. jemenitica*. During the initial learning trial, neither the control nor the treated bees exhibited PER. During the second and third learning trials, the PER was dependent on the IM concentration, with LC_30_ causing the greatest reduction, followed by LC_20_ and LC_10_, while the control bees exhibited the highest PER (Figure 6a and Table S4). Memory formation was significantly decreased with all tested concentrations of IM after 2, 12, and 24 h compared to the control group (Figure 6b and Table S4).

#### 3.2.2. Oral Exposure of IM and Associative Learning

**Figure 7:**
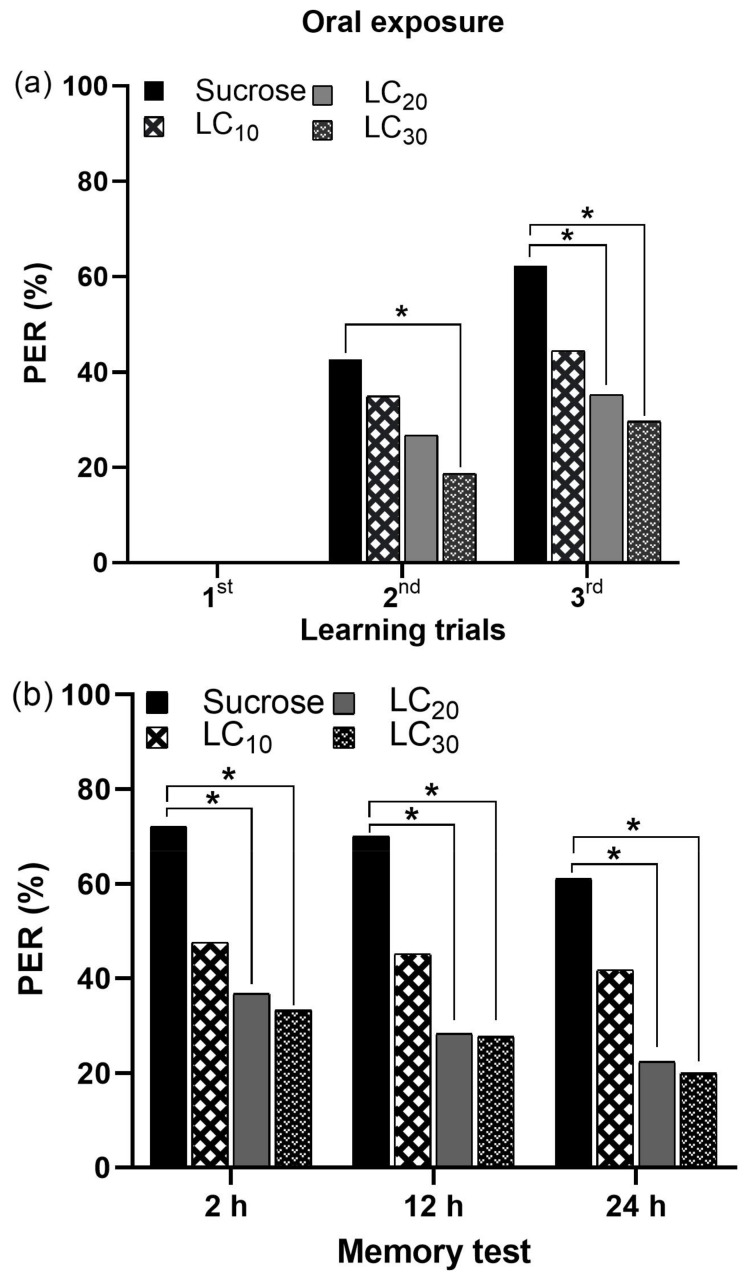
Proboscis extension response (PER) of bees during (**a**) learning trials and (**b**) memory tests following oral exposure to various sublethal concentrations of IM. Asterisk (*) indicate a significant difference between the control and IM-treated bees (Fisher’s exact test/χ^2^ test; * *p* < 0.05).

The oral ingestion of sublethal concentrations of IM (LC_10_ = 2.45 ppm, LC_20_ = 4.04 ppm, and LC_30_ = 5.78 ppm) adversely affected the olfactory learning and memory of *A. m. jemenitica*. During the initial learning trial, neither the control nor the treated bees exhibited PER. During the second and third learning trials, the PER was dependent on the IM concentration, with LC_30_ causing the greatest reduction, followed by LC_20_ and LC_10_, while the control bees exhibited the highest PER (Figure 7a and Table S5). Memory formation was significantly decreased with all tested concentrations of IM after 2, 12, and 24 h compared to control group (Figure 7b and Table S5).

Although the combination index (CI) values for the mortality data after oral exposure suggest an antagonistic effect (Table 1 and Figure 4), resulting in a relatively lower bee mortality compared to topical exposure (Figure 5), IM was equally effective via either exposure route in significantly reducing the PER during both the learning and memory tests (Supplementary Figure S1a,b). Furthermore, the comparison of the cumulative mean PER in response to IM, deltamethrin, and acetamiprid during the learning and memory phases revealed that IM was equally effective in reducing PER through oral and topical exposure, suggesting no antagonistic or synergistic effects between the two exposure routes (Supplementary Figure S1c).

## 4. Discussion

The current study focused on the combined mixture of acetamiprid and deltamethrin—belonging to two different classes (neonicotinoids and pyrethroids, respectively)—and found that the mixture of these insecticides exerted a substantial toxic effect against honey bees^’^ survival and cognitive performance related to learning and memory formation. These findings regarding bee survival align with existing evidence on insecticides including neonicotinoids and pyrethroids, indicating that diverse mixtures of insecticides can induce enhanced toxicity in honey bees [78]. Furthermore, the combination index (CI) utilized in the current study revealed the nature of interaction among insecticides in the mixture, indicating whether these compounds acted synergistically, antagonistically, or additively [67,71,79,80]. This research highlights the importance to assess the combined effects of insecticide mixtures rather than focusing solely on individual insecticides.

Our data revealed that the insecticide mixture (IM) of deltamethrin and acetamiprid resulted in significant bee mortality through both topical and oral exposure routes, with topical exposure proving to be more effective in inducing a higher mortality. Oral exposure significantly reduced the food intake, and this decline in consumption progressively increased with higher IM concentrations. The LC_50_ values 24 h post treatment were 12.24 ppm for topical exposure and 10.45 ppm for oral exposure. Learning and memory formation in bees were significantly impaired by exposure to sublethal concentrations (LC_10_, LC_20_, and LC_30_) of IM through both exposure routes.

### 4.1. Insecticide Mixture (IM) and Bee Mortality

The topical and oral application of the neonicotinoid–pyrethroid mixture (acetamiprid–deltamethrin) resulted in significant mortality in *A. m. jemenitica* at different post-treatment time points. The analysis of CI revealed synergistic effects between the insecticides during topical exposure and antagonistic effects during oral exposure to IM. The higher mortality observed with the topical application of IM could be attributed to this synergistic effect. Moreover, in topically exposed bees, the mortality rates significantly differed between the IM and individual insecticides. Conversely, oral feeding of IM resulted in a relatively lower mortality in the treated bees compared to the topically exposed bees. This was likely due to the antagonistic effect between the insecticides. Interestingly, despite the antagonism observed during oral exposure, no significant differences were found in the mortality rates of bees induced by orally administered IM and those treated with individual insecticides. Taken together, IM (acetamiprid–deltamethrin) had the potential to cause prominent bee mortality through both topical and oral exposure routes.

There is a disparity among studies regarding the impact of various insecticide mixtures on bee mortality. Some studies report higher mortality rates with IM compared to individual insecticides, while others find no significant differences, highlighting the varied interactions between insecticides across different experimental setups. Numerous studies investigated bee mortality across different insecticides, fungicides, and bee species. The combination of fipronil, thiacloprid, and *Nosema ceranae* infection administrated orally with a 50% sucrose solution resulted in high mortality in *A. mellifera* [81]. Likewise, the topical application of an insecticide–fungicide (chlorantraniliprole–propiconazole) combination was highly toxic to adult honey bee workers compared to those exposed to the insecticide alone [82]. The chronic oral exposure of a mixture of neonicotinoid–fungicide (acetamiprid–propiconazole) also significantly increased forager bee (*A. cerana*) mortality to 50% after 4.8 days [83]. Fungicides can interact synergistically, considerably amplifying the toxicity of neonicotinoids and pyrethroids towards bees [5]. A mixture of clothianidin (neonicotinoid) and λ-cyhalothrin (pyrethroids) demonstrated synergistic effects influencing the survivorship of adult *A. mellifera* [84]. Similarly, mixtures of neonicotinoid–organophosphate (thiamethoxam–chlorpyrifos) reveal a synergistic effect for the mortality of bumblebees (*Bombus terrestris*) [73]. The combination of thiamethoxam (a neonicotinoids), pyrethroids (zeta-cypermethrin, cyfluthrin, and permethrin), and other insecticides exhibits synergistic action with enhanced toxicity for honey bees during oral feeding tests [78]. Neonicotinoids (thiamethoxam and clothianidin) and *Varroa* mites interact synergistically to negatively affect overwintering *A. mellifera*‘s survival after oral feeding of contaminated pollen paste [85]. The mixtures of acetamiprid with glyphosate or tebuconazole, as well as ternary mixtures, demonstrate greater toxicity to bees than individual pesticides after oral administration [86]. Chronic oral exposure to IM (imidacloprid and difenoconazole) was also shown to cause higher mortality in *A. mellifera* compared to exposure to individual insecticides [56].

The oral administration of a thiamethoxam and cypermethrin mixture showed no synergism, yielding mortality effects comparable to the individual pesticides [87]. This is in consistent with our results, where the acetamiprid–deltamethrin combination also yielded mortality comparable to that of the individual insecticides. Likewise, imidacloprid combined with coumaphos (fungicide) did not significantly increase mortality compared to the control or individual pesticides [88].

It is interesting that, despite the CI suggesting antagonism for IM during oral exposure, IM (acetamiprid–deltamethrin) resulted in notable bee mortality. The comparative analysis of mortality rates further indicates that IM caused a relatively lower mortality; however, the differences in mortality were non-significant compared to the mortality caused by individual insecticides following oral exposure. Moreover, our results revealed that a low sublethal concentration of IM applied topically could induce mortality similar to that caused by the oral administration of a higher sublethal concentration. This could be attributed to enzymes in the honey bee midgut [89] and microbiota that detoxify and degrade insecticides after feeding [90,91]. However, after topical exposure, IM is directly absorbed into the bee’s thorax by passing the detoxification and resulting in high mortality at low concentrations. In contrast, oral exposure requires higher concentrations to achieve a similar degree of mortality.

The oral feeding of varying concentrations of IM (acetamiprid–deltamethrin) significantly reduced food consumption rates in *A. m. jemenitica*, with the intake decreasing as the IM concentration increased. In agreement, exposure to binary (AT, GA, and GT) and ternary (GTA) mixtures of acetamiprid (A), glyphosate (G), and tebuconazole (T) considerably reduced food intake in *A. m. carnica* compared to the control and individual pesticide groups [86]. The oral administration of imidacloprid (a neonicotinoid) led to a reduction in syrup consumption, thoracic temperature, and bee activity of the solitary bee species, *Osmia bicornis* L. [92]. In contrast, sucrose consumption over 24 h showed no significant differences between the control group and those fed a mixture of imidacloprid and coumaphos [88].

The IM (acetamiprid–deltamethrin) was significantly more toxic to *A. m. jemenitica* via the topical route than through oral exposure. Studies have reported variability in insecticide effects, with some indicating higher mortality after topical exposure, others showing greater mortality from the oral route, and some finding no difference between the two methods. Our results revealed that IM administered through the topical route was significantly more effective in causing a high mortality in *A. m. jemenitica* than oral exposure. This finding aligns with Shah et al. [93], who reported greater mortality in *A. mellifera* following contact exposure compared to oral exposure to acetamiprid. However, no significant differences were found between oral and topical exposure to deltamethrin [93].

### 4.2. IM-Induced Changes in Cognitive Performance of Bees

The proboscis extension response (PER) is a behavioral assay used to assess honey bees’ olfactory learning and memory. It is a reliable indicator of cognitive function, particularly useful for studying the impact of environmental stressors, such as pesticides [74]. Reduced PER responses suggest impaired learning and memory, often indicating neurotoxic effects from chemicals [41,74,75]. The IM (acetamiprid–deltamethrin) at sublethal concentrations (LC_10_, LC_20_, and LC_30_) negatively affected learning and impaired memory capabilities in *A. m. jemenitica*. The reduction in PER was concentration-dependent, with LC_30_ resulting in the highest reduction during the learning and memory trials, followed by LC_20_ and LC_10_ and the control bees, which exhibited the highest PER. IM-treated bees showed a reduced PER during the learning trials and memory formation after 2, 12, and 24 h compared to the control bees, following both topical and oral administration. While the effects of insecticides from various groups on learning and memory are well documented, research on the effects of insecticide mixtures remains limited. The existing literature provides examples of combinations of insecticides with fungicides or miticides that affect honey bees. Among the available research, variability in outcomes is apparent, with some studies demonstrating notable effects of IM on cognitive performance in honey bees, while others report no effect. A neonicotinoid–fungicide (thiamethoxam–carbendazim) mixture was shown to significantly decrease the PER in *A. mellifera* at 24 and 72 h post treatment [94]. Conversely, the fungicide–neonicotinoid combination administered orally shows no adverse effects on sucrose responsiveness and learning performance in *A. mellifera* [95]. The imidacloprid–coumaphos (neonicotinoid–fungicide) combination has been shown to negatively affect bees’ learning and memory, impairing their memory formation abilities [88]. The combination of miticide–neonicotinoid (thymol–imidacloprid treated as plastic strips between brood frames), diminishes the performance of *A. mellifera* in visual learning tests [96]. In contrast, the oral administration of a mixture of four organophosphates (diazinon, malathion, profenofos, and chlorpyrifos) shows no effect on *A. mellifera* during learning and memory tests [97].

The discrepancy between the higher mortality induced by IM in the topical exposure groups compared to oral exposure, alongside an equally comparable performance in the PER tests following oral exposure, can be explained by the differing modes of action and absorption rates of the insecticides via the two exposure routes. Topical exposure typically results in a more immediate and concentrated absorption of the insecticide through the cuticle, leading to a higher toxicity and, consequently, a greater mortality. In contrast, oral exposure involves ingestion and digestion, which may result in the delayed onset of toxicity due to the slower absorption and metabolism of the insecticide. This difference in absorption and metabolism rates likely accounts for the observed variation in mortality despite comparable sublethal effects, such as impaired learning and memory, without any evidence of antagonistic or synergistic effects, as seen in the PER tests.

The data from the current research provide evidence of a significant reduction in the PER of honey bees during the learning and memory formation stages after treatment with the IM (neonicotinoid–pyrethroids), administered through both oral and topical routes.

## 5. Conclusions

This research highlights the significant detrimental effects of a mixture of acetamiprid and deltamethrin insecticides on the mortality and cognitive functions, especially learning and memory, of *A. m. jemenitica*. Notably, the IM significantly influenced the learning and memory capacities of bees when administered through topical and oral exposure routes. The mixture demonstrated particularly severe effects through topical exposure, resulting in a higher mortality compared to oral exposure, while also significantly reducing food intake during oral ingestion. The impairment of cognitive capacities, particularly learning and memory, associated with both exposure routes emphasized the pervasive risks posed by the IM to honey bee populations. These findings point to an urgent need for more comprehensive studies on the sublethal effects of insecticide mixtures on honey bee health and behavior to inform better management practices and policies aimed at protecting these critical pollinators.

## Figures and Tables

**Table 1:** Combination index (CI) and insecticide mixture (IM).

Exposure Type	Combination Index (CI) *	
Topical	0.43	Synergistic
Oral	1.40	Antagonistic

* Additive (CI = 1), antagonistic (CI > 1), and synergistic (CI < 1).

**Table 2:** Lethal and sublethal concentrations of insecticide mixture 24 h post exposure.

Exposure Type	Lethal Concentration (ppm)		Sublethal Concentration (ppm)		
	LC_50_	LC_90_	LC_10_	LC_20_	LC_30_
Topical	12.24	40.04	3.75	5.63	7.54
Oral	10.45	44.46	2.45	4.04	5.78

## Data Availability

All data are provided within this article.

## Supplementary Materials

The following supporting information can be downloaded at: https://www.mdpi.com/article/10.3390/biology14020147/s1, Figure S1: Comparison of PER in response to acetamiprid-deltamethrin mixture and individual insecticides; Table S1: IM topical exposure and bee mortality (For Figure 1); Table S2: IM oral exposure and bee mortality (For Figure 2); Table S3: Food consumption after IM oral exposure (For Figure 3); Table S4: IM topical exposure and PER (For Figure 6); Table S5: IM oral exposure and PER (For Figure 7).
