# Supplementary material for: Binary Mixture of Neonicotinoid–Pyrethroid Insecticide: Impact on Survival, Cognitive Learning, and Memory in Apis mellifera jemenitica
Source: Biology (Basel). 2025 Jan 30;14(2):147. doi: 10.3390/biology14020147 (PMC11851376; doi:10.3390/biology14020147)
Supplement: Supplementary file 1 [file biology-14-00147-s001.zip › Sublementary Tables and Figures.pdf]

**Mohamedazim I. B. Abuagla [1], Javaid Iqbal [1], Hael S. A. Raweh [1], Abdelsalam S. A. Abdelaziz [2] and Abdulaziz S. Alqarni [1,*]**

# Supplementary Tables

## SUPPLEMENTARY TABLE S1: IM topical exposure and bee mortality (For Figure 1)

### Table S1-A: ANOVA for bee morality at individual times

| Treatment (IM-Concentrations)* | Time | df | Sum of Squares | Mean Square | F Value | *P* |
|---|---|---|---|---|---|---|
| Between Concentrations | 4 h | 4 | 19430 | 4857 | 132.48 | <.0001 |
| Between Concentrations | 12 h | 4 | 26920 | 6730 | 175.57 | <.0001 |
| Between Concentrations | 24 h | 4 | 29030 | 7257 | 435.45 | <.0001 |
| Between Concentrations | 48 h | 4 | 30020 | 7505 | 409 | <.0001 |

*C1(control) = 0 ppm; C2 = 24.60 ppm; C3 = 49.50 ppm; C4 = 98.25 ppm; C5 = 196.50 ppm

### Table S1-B: LSD All-Pairwise Comparisons

| Time | Treatment (IM-Concentrations)* | Mean Corrected Mortality (%) | Significance ($p \leq 0.05$) |
|---|---|---|---|
| 4 h | C1 | 0.00 | d |
| | C2 | 57.50 | c |
| | C3 | 70.00 | b |
| | C4 | 80.00 | a |
| | C5 | 88.00 | a |
| 12 h | C1 | 0.00 | d |
| | C2 | 70.00 | c |
| | C3 | 88.00 | b |
| | C4 | 95.00 | ab |
| | C5 | 100 | a |
| 24 h | C1 | 0.00 | d |
| | C2 | 75.00 | b |
| | C3 | 97.50 | a |
| | C4 | 97.50 | a |
| | C5 | 100 | a |
| 48 h | C1 | 0.00 | d |
| | C2 | 78.00 | b |
| | C3 | 100 | a |
| | C4 | 100 | a |
| | C5 | 100 | a |

*C1(control) = 0 ppm; C2 = 24.60 ppm; C3 = 49.50 ppm; C4 = 98.25 ppm; C5 = 196.50 ppm

Mohamedazim I. B. Abuagla [1], Javaid Iqbal [1], Hael S. A. Raweh [1], Abdelsalam S. A. Abdelaziz [2] and Abdulaziz S. Alqarni [1,*]

**SUPPLEMENTARY TABLE S2: IM oral exposure and bee mortality (For Figure 2)**

**Table S2-A: ANOVA for bee morality at individual times**

| Treatment (IM-Concentrations)* | Time | df | Sum of Squares | Mean Square | F Value | *P* |
|---|---|---|---|---|---|---|
| Between Concentrations | 4 h | 4 | 11000 | 2750 | 41.25 | <.0001 |
| Between Concentrations | 12 h | 4 | 21020 | 5255 | 34.65 | <.0001 |
| Between Concentrations | 24 h | 4 | 22480 | 5620 | 38.76 | <.0001 |
| Between Concentrations | 48 h | 4 | 24950 | 6237.5 | 35.64 | <.0001 |

*C1 (control) = 0 ppm; C2 = 5.65 ppm; C3 = 11.30 ppm; C4 = 22.65 ppm; C5 = 45.30 ppm

**Table S2-B: LSD All-Pairwise Comparisons**

| Time | Treatment (IM-Concentrations)* | Mean Corrected Mortality (%) | Significance *(p ≤0.05)* |
|---|---|---|---|
| 4 h | C1 | 0.00 | d |
| | C2 | 15.00 | c |
| | C3 | 30.00 | b |
| | C4 | 35.00 | b |
| | C5 | 70.00 | a |
| 12 h | C1 | 0.00 | d |
| | C2 | 22.00 | c |
| | C3 | 45.00 | b |
| | C4 | 60.00 | b |
| | C5 | 95.00 | a |
| 24 h | C1 | 0.00 | d |
| | C2 | 35.00 | c |
| | C3 | 52.50 | bc |
| | C4 | 70.00 | b |
| | C5 | 100 | a |
| 48 h | C1 | 0.00 | d |
| | C2 | 37.00 | c |
| | C3 | 67.50 | b |
| | C4 | 82.50 | ab |
| | C5 | 100 | a |

*C1 (control) = 0 ppm; C2 = 5.65 ppm; C3 = 11.30 ppm; C4 = 22.65 ppm; C5 = 45.30 ppm

**SUPPLEMENTARY TABLE S3:** **Food consumption after IM oral exposure (For Figure 3)**

**Table S3-A: ANOVA for food consumption**

| Treatment (IM-Concentrations)* | df | Sum of Squares | Mean Square | F Value | P |
|---|---|---|---|---|---|
| Between Concentrations | 4 | 2332 | 583 | 5.40 | <.0.0067 |

*C1 (control) = 0 ppm; C2 = 5.65 ppm; C3 = 11.30 ppm; C4 = 22.65 ppm; C5 = 45.30 ppm

**Table S3-B: LSD All-Pairwise Comparisons**

| Treatment (IM-Concentrations)* | Mean (% Food Consumption) | Significance |
|---|---|---|
| C1 | 100 | a |
| C2 | 83.00 | b |
| C3 | 71.00 | c |
| C4 | 62.00 | c |
| C5 | 44.00 | d |

*C1 (control) = 0 ppm; C2 = 5.65 ppm; C3 = 11.30 ppm; C4 = 22.65 ppm; C5 = 45.30 ppm

**Mohamedazim I. B. Abuagla [1], Javaid Iqbal [1], Hael S. A. Raweh [1], Abdelsalam S. A. Abdelaziz [2] and Abdulaziz S. Alqarni [1,*]**

**SUPPLEMENTARY TABLE S4:** **IM topical exposure and PER (For Figure 6)**

| Learning and memory formation | | | Fisher's exact test/ $\chi 2$ test value | Significance *(p<0.05)* |
|---|---|---|---|---|
| Learning Trials | 2nd | C1 vs C2 | 0.512 | n.s |
| | | C1 vs C3 | 0.044 | Sig. |
| | | C1 vs C4 | 0.033 | Sig. |
| | | C2 vs C3 | 0.712 | n.s |
| | | C2 vs C4 | 0.572 | n.s |
| | | C3 vs C4 | 0.844 | n.s |
| | 3rd | C1 vs C2 | 0.542 | n.s |
| | | C1 vs C3 | 0.042 | Sig. |
| | | C1 vs C4 | 0.041 | Sig. |
| | | C2 vs C3 | 0.081 | n.s |
| | | C2 vs C4 | 0.150 | n.s |
| | | C3 vs C4 | 1.000 | n.s |
| Memory | 2 h | C1 vs C2 | 0.024 | Sig. |
| | | C1 vs C3 | 0.033 | Sig. |
| | | C1 vs C4 | 0.016 | Sig. |
| | | C2 vs C3 | 1.000 | n.s |
| | | C2 vs C4 | 0.856 | n.s |
| | | C3 vs C4 | 0.854 | n.s |
| | 12h | C1 vs C2 | 0.043 | Sig. |
| | | C1 vs C3 | 0.039 | Sig. |
| | | C1 vs C4 | 0.015 | Sig. |
| | | C2 vs C3 | 0.840 | n.s |
| | | C2 vs C4 | 0.680 | n.s |
| | | C3 vs C4 | 0.832 | n.s |
| | 24 h | C1 vs C2 | 0.280 | n.s |
| | | C1 vs C3 | 0.042 | Sig. |
| | | C1 vs C4 | 0.010 | Sig. |
| | | C2 vs C3 | 0.677 | n.s |
| | | C2 vs C4 | 0.675 | n.s |
| | | C3 vs C4 | 1.000 | n.s |

*C1 (control) = Acetone; C2 = $LC_{10}$ (3.75 ppm); C3 = $LC_{20}$ (5.63 ppm); C4 = $LC_{30}$ (7.54 ppm). Sig. stands for Significant; n.s stands for non-significant.

**SUPPLEMENTARY TABLE S5: IM oral exposure and PER (For Figure 7)**

| Learning and memory formation | | | Fisher's exact test/ χ2 test value | Significance *(p<0.05)* |
|---|---|---|---|---|
| Learning Trials | 2nd | C1 vs C2 | 0.558 | n.s |
| | | C1 vs C3 | 0.180 | n.s |
| | | C1 vs C4 | 0.033 | Sig. |
| | | C2 vs C3 | 0.456 | n.s |
| | | C2 vs C4 | 0.117 | n.s |
| | | C3 vs C4 | 0.380 | n.s |
| | 3rd | C1 vs C2 | 0.270 | n.s |
| | | C1 vs C3 | 0.043 | Sig. |
| | | C1 vs C4 | 0.024 | Sig. |
| | | C2 vs C3 | 0.750 | n.s |
| | | C2 vs C4 | 0.200 | n.s |
| | | C3 vs C4 | 0.340 | n.s |
| Memory | 2 h | C1 vs C2 | 0.161 | n.s |
| | | C1 vs C3 | 0.026 | Sig. |
| | | C1 vs C4 | 0.018 | Sig. |
| | | C2 vs C3 | 0.618 | n.s |
| | | C2 vs C4 | 0.214 | n.s |
| | | C3 vs C4 | 0.457 | n.s |
| | 12h | C1 vs C2 | 0.148 | n.s |
| | | C1 vs C3 | 0.005 | Sig. |
| | | C1 vs C4 | 0.012 | Sig. |
| | | C2 vs C3 | 0.460 | n.s |
| | | C2 vs C4 | 0.289 | n.s |
| | | C3 vs C4 | 0.741 | n.s |
| | 24 h | C1 vs C2 | 0.245 | n.s |
| | | C1 vs C3 | 0.006 | Sig. |
| | | C1 vs C4 | 0.019 | Sig. |
| | | C2 vs C3 | 0.146 | n.s |
| | | C2 vs C4 | 0.104 | n.s |
| | | C3 vs C4 | 0.832 | n.s |

*C1 (control) = Sucrose; C2 = $LC_{10}$ (2.45 ppm); C3 = $LC_{20}$ (4.04 ppm); C4 = $LC_{30}$ (5.78 ppm). Sig. stands for Significant; n.s stands for non-significant.

# Supplementary Figure

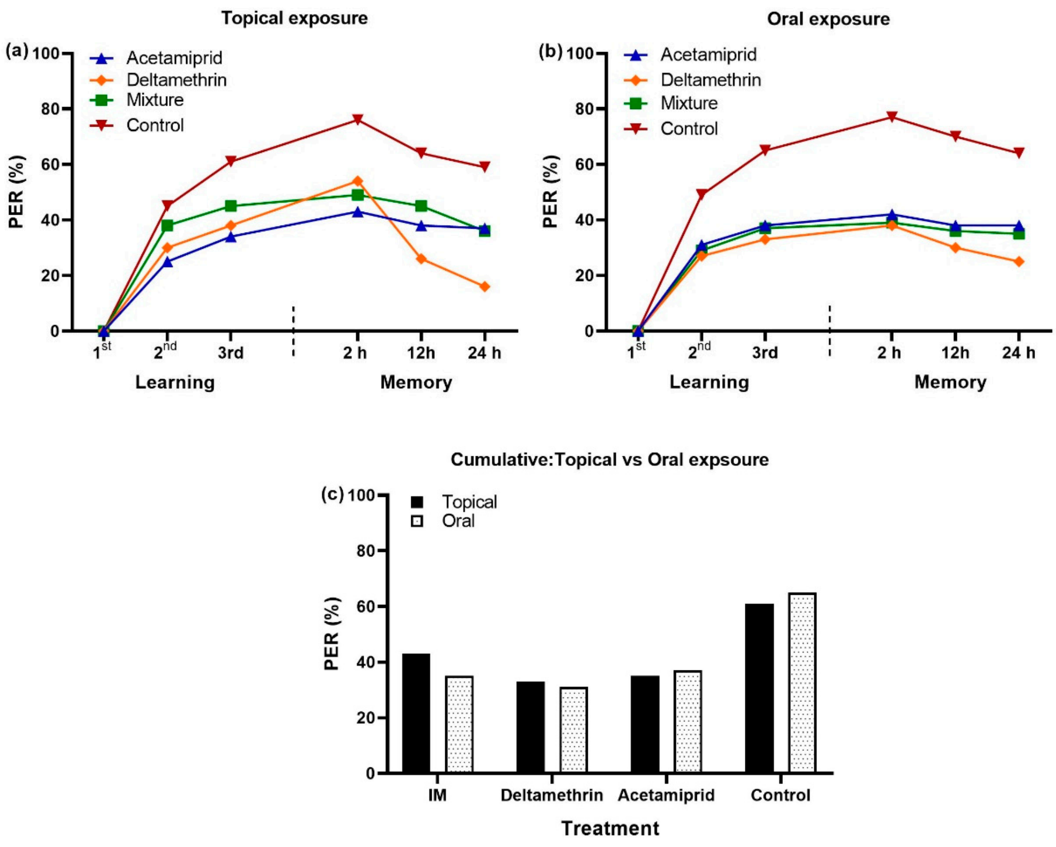


**Figure S1.** Comparison of PER in response to acetamiprid-deltamethrin mixture and individual insecticides: a) Topical exposure, b) Oral exposure c) Cumulative comparison: Topical Vs. oral exposure.
